# Advantages of Using 3D Intraoperative Ultrasound and Intraoperative MRI in Glioma Surgery

**DOI:** 10.3389/fonc.2022.925371

**Published:** 2022-06-03

**Authors:** Yuanzheng Hou, Jie Tang

**Affiliations:** Department of Neurosurgery, Xuanwu Hospital, Capital Medical University, Beijing, China

**Keywords:** intraoperative ultrasound, 3D intraoperative ultrasound, intraoperative MRI, glioma, surgery

We read with great interest the recent article by Bastos et al (1):” Challenges and Opportunities of Intraoperative 3D Ultrasound with Neuronavigation in Relation to Intraoperative MRI”.

In this study, the authors reported their experiences using both 3D intraoperative ultrasound(iUS) and intraoperative MRI (iMRI) in 23 glioma surgeries. In 65% of patients, the tumors are located near the eloquent areas. 43% were recurrent cases. Three 3D iUS scans were routinely performed before and after dural opening and before the iMRI scan. More 3D iUS scans might be added if necessary. After tumor resection, one or two iMRI scans were performed to confirm the residual tumor. Multimodule neuro-navigation and electrophysiological monitoring techniques were also utilized during surgery. After surgery, 53% of patients achieved gross total resection. 21% of patients had temporary neurological deficits. None of them were permanent. In 82% of patients, the results of iUS were consistent with iMRI findings. While in the other four recurrent cases, the image quality of iUS was poor for the authors to decide.

Three cases were presented in the paper. Case one was a small tumor located just before the precentral gyrus. IUS showed an apparent brain shift just after the dural opening. This information helped the surgeon choose the right site without causing injuries to the precentral gyrus. Case two was a giant left temporal glioma surrounded by bunches of fiber tracts. Multiple iUS scans were utilized to assess the resection process serially and helped the surgeon find the residual tumor. Case three was a low-grade glioma growing along the cingulate gyrus. 3D iUS helped the surgeon choose a safe corridor between functional areas and delineate the anatomical structures surrounding the tumor. The small residual tumor was found by iUS and confirmed by iMRI. The authors concluded that using 3D iUS in relation to iMRI had practical benefits for glioma surgeries and challenges.

We agree with the authors’ opinion regarding the advantages and limitations of the iUS and iMRI. Combining these two techniques in glioma surgery seems reasonable and promising. Their conclusions are well supported by the data and illustrated cases. In addition to congratulating the authors, we would like to point out a few issues.

The initial surgical treatment of glioma aims to obtain the maximal extent of resection (EOR) while preserving neurological function (2, 3). However, surgeon assessment of EOR based on visualization is prone to error, even when performed by experienced clinicians (4). Therefore, intraoperative imaging techniques such as iUS and iMRI were introduced into glioma surgery to achieve reliable resection control.

iUS has been used in neurosurgery since the 1980s and is significantly correlated with preoperative MRI assessment of tumor volume (5–7). Mounting evidence also demonstrates the beneficial effects of ioUS for resection control in glioma surgery (8–10). The notable advantage of iUS is that it provides nearly real-time imaging without interfering with the surgical workflow (11). The iUS information can be used to modify the surgical strategy intraoperatively (9). However, the major drawback of iUS is its steep learning curve and its prone to artifacts (12).

iMRI system was introduced into neurosurgery in the 1990s. Modern iMRI generates excellent images with few artifacts (13). Accumulated evidence shows that iMRI increases the EOR and gross total resection (GTR) rate in glioma surgery and improves progression-free survival and overall survival (2, 3). However, the major drawback of iMRI is the interruption of the operative flow. In clinical practice, most centers prefer to perform iMRI scans at the end of the resection process for final control (14–16). Due to the limited number of scans, surgical decisions based on iMRI are limited.

The complementary characteristics of iMRI and iUS raise the possibility of integrating them into glioma surgery. Firstly, iUS can generate nearly real-time intraoperative images (11). With iUS, surgeons can adjust their surgical strategy on time. Secondly, iUS has high sensitivity and specificity for detecting tumor borders in the initial assessment. If the histopathological results are taken as the gold standard. The sensitivity and specificity of iUS are 95% for the glioblastoma and 72%–86%, and 75%–100% for the low-grade glioma (17). Additionally, iUS is better than iMRI at distinguishing tumors from brain tissue and edema (5, 7, 17). Bastos’s study also showed that iUS could delineate detailed structures such as sulci and gyri, which are obscure on iMRI, and compensate for brain shift just after dural opening. Thus, iUS may provide valuable additional or complementary information at the beginning of the resection. Thirdly, even though the sensitivity of iUS is acceptable during surgery (87%), it decreases to 20% at the end of resection (6, 12, 18). Therefore, iUS might be more appropriate for monitoring the resection process than for performing the final resection control. By further using iMRI to perform the final resection control, the whole tumor resection process might be controlled by an integrated approach that capitalizes on the advantages of both imaging modalities.

Bastos’s study supported the feasibility of integrating these two intraoperative imaging techniques. iUS provided much complementary intraoperative information to help the surgeon identify brain shift (case one), perform resection control serially (case two), and choose a safe surgical corridor (case three). On the other hand, iMRI approved residual tumors that were not detected by iUS in 8.7% of patients. In the end, they achieved good surgical results by combine using these two techniques.

We commend the authors for their interesting study elucidating the utility of 3D iUS together with iMRI. This is a valuable exploration of the surgical strategy of glioma. However, it would be more helpful if the following questions could be addressed in their future studies.

The training level of the team members in the iUS and iMRI was not mentioned in the paper. The learning curve of understanding the ultrasound image was steep, especially in the intraoperative circumstance (19). The 3D iUS helps a lot by providing coaxial preoperative MRI images. However, the apparent deformation of the brain tissue with the resection process can limit the helpfulness of the coaxial MRI images. There is still a need for an understanding of the echogenicity of various brain structures, both normal and pathological (19). Other tasks, such as acquiring high-quality 3D iUS data, identifying the residual tumor from the hyperechoic rim surrounding the resection cavity, and cross-referencing between iUS and iMRI images, also need adequate training. So, the training level of the team members had a marked impact on the final results. It would be beneficial to other centers interested in integrating iUS with iMRI if the author could provide additional suggestions about the training requirements.

A 100% concordance rate between 3D iUS and iMRI was reported in the study, which is different from those reported in the literature (20). Considering only newly diagnosed gliomas, iUS’s sensitivity and specificity in this study were 100% when finding residual tumors following tumor resection. While in the literature, the sensitivity was around 26% towards the end of surgery (6, 21). We also noticed that 30% of tumors were deep-seated and 65% were recurrent. Our experience and literature reports indicate that the image quality of the iUS is more likely to have artifacts in the deep resection cavity or after radiation therapy (22). It would be more helpful if the authors could elaborate on their experiences in improving the specificity and sensitivity of iUS.

The complexity of introducing iUS into iMRI-assisted surgery was not mentioned and assessed in the study. To our opinion, this is a question that should be considered before using this technique. Using the modern high field iMRI system has inevitably increased the system complexity and the surgical time and cost (15). Extensive preparation procedures, careful safety checks, pre-closing, and re-draping process before and after each iMRI scan are all complicated and have low error tolerances. In this circumstance, is it worth introducing a new intraoperative imaging technique into the system? Would the further increased system complexity reverse the beneficial effects of this technique in the large cohort? These are still open questions needed to be answered in the future. On the other hand, we noticed that iUS demonstrated a high specificity when identifying the residual tumor in this study. Whether the using of iUS could decrease the need for multiple iMRI scans? The authors did not give specific data about the number of iMRI scans. While In the reported iMRI case series, around 46% (26.1–52.2%) (14–16, 23) patients needed multiple iMRI scans. Reducing the number of iMRI scans also might effectively decrease the complexity of the iMRi-assisted surgeries. It would be much more valuable if the authors could provide the data about the changes in iMRI scan number, surgical time, and financial cost of this group of patients, compared with only iMRI cases.

The study enrolled patients with a higher risk of postoperative neurological complications. In 65% of selected patients, the tumors were located near the eloquent areas. The functional result after surgery was also excellent compared with the literature data (20, 24). Previous iUS series reported that surgery-induced permanent neurological deficits occurred in 8%–13% of patients (25, 26). However, the use of the iUS was not clearly described in how it contributed to the surgical outcome in the study. In illustrated cases, the pre-resection iUS helped the surgeon identify the brain shift and choose safe surgical corridors. When approaching the eloquent structures, the serial checks of iUS allowed the surgeon to control the process more precisely. Compared with a minimal number of iMRI scans, iUS scans provided more intraoperative information for the surgeon to modify the surgical strategy. These facts all might contribute to the protection of neurological function. However, their beneficial effects were all indirect. High-level evidence is still lacking concerning whether iUS might reduce neurological deficits after surgery. Unfortunately, the authors did not summarize and discuss this question in detail. Furthermore, the multimodule neuro-navigation and neurophysiological monitor techniques were also utilized in these high-risk patients. In case one, continuous subcortical motor mapping and serial iUS scan were used when approaching the motor pathway. However, the authors did not specify the criteria they used to stop resection besides the fibers: iUS or current threshold? While in case two, a partial tumor was left according to the iUS and fiber tracking result instead of mapping results. Using the iUS could generate more information, allowing for more informed and, thus, higher-quality decisions. Nevertheless, how to combine iUS with these technologies in a reasonable way remains an open question.

The study did not explicitly discuss the advantages of combining iUS with iMRI for intraoperative decision-making. A significant benefit of intraoperative imaging is that it aids in intraoperative decision-making (20). With iMRI, a reliable assessment of the need for further resection can be made. The rates of further resection range from 26.1% to 52.2% in the reported iMRI case series (20, 27). Data from this study well supported this iMRI’s advantage over the iUS for final resection assessment. In 17% (4/23) patients, the iUS cannot reach a reliable judgment on the residual tumor. Thus, iMRI could effectively complement ioUS in terms of resection control, leading to a better GTR. On the other hand, the advantage of iUS is its ability to provide real-time information. In all three illustrated cases, surgeons modified their surgical strategy intraoperatively based on iUS findings at the beginning and during resection. Therefore, iUS effectively complement iMRI, given its the limited number of intraoperative scans. Nonetheless, the author did not analyze in detail the intraoperative decision-making based on iUS and iMRI findings. The benefits of incorporating iUS and iMRI were not clearly outlined in the paper. According to our experience, iUS can help the intraoperative decision-making process in many other situations, such as large glioma with irregular shapes, glioma with unclear boundaries on iMRI images, or safely approaching the eloquent structures. It would be more informative if the authors could summarize their intraoperative decision-making processes according to iUS and iMRI.

## Author Contributions

All authors have made a substantial, direct and intellectual contribution to the work, and approved it for publication.

## Conflict of Interest

The authors declare that the research was conducted in the absence of any commercial or financial relationships that could be construed as a potential conflict of interest.

## Publisher’s Note

All claims expressed in this article are solely those of the authors and do not necessarily represent those of their affiliated organizations, or those of the publisher, the editors and the reviewers. Any product that may be evaluated in this article, or claim that may be made by its manufacturer, is not guaranteed or endorsed by the publisher.
